# Effect of kerosene combustion atmosphere on the mild steel oxide layer

**DOI:** 10.1038/s41598-021-04377-3

**Published:** 2022-01-10

**Authors:** Dongbai Xie, Hao Hong, Shuwang Duo, Qiang Li

**Affiliations:** 1 Faculty of Electro-Machical Information, Weifang University of Science and Technology, Shouguang, 262700 China; 2 Jiangxi Key Laboratory of Materials Surface Engineering, Jiangxi Science and Technology Normal University, Nanchang, 330013 China

**Keywords:** Materials science, Structural materials

## Abstract

In arson cases, accelerants were usually used by criminals to achieve the purpose of rapid arson. Therefore, fire investigators aim to determine whether accelerants was used in the fire scene. Metallic material has to react with corrosive gas around it at high temperature and the oxidation products may store the information of reactants. Accelerants present in fire scenes impart some oxidative characteristics on metallic materials. The aim of this work is to figure out the possibility to identify the presence of accelerant in a fire according to the oxidation patterns of metallic material. This paper researched the oxidation behavior of mild steel at high temperature in a simulated flame environment. The surface morphological and cross-sectional microstructural features of the samples were characterized by X-ray diffractions, X-ray photoelectron spectroscopy and scanning electron microscopy with energy-dispersive spectroscopy analysis after oxidation. The carbon in the combustion atmosphere had a carburizing effect on the metal oxide layer. It was mostly C–C, C–O and C=O of organic matter could be used as in fire investigation. Various oxidizing atmosphere composite systems promote the formation of metal oxide layers. And bidirectional oxidation mode in the oxide layer further accelerates the oxidation rate. The (wustite) FeO phase was not found in the oxide layer because of the strong oxidation of the combustion atmosphere. These results offer complementary information in fire characteristics, which combining the characterization of surface scale with traditional chemical analysis of recovering ignitable liquid residues from fire debris are expected to offer crucial information for determining the presence of combustion accelerants at a fire scene.

## Introduction

Scientific based evidence is needed in court trials. The analysis of fire debris evidence might offer crucial information to a forensic investigation, when for instance, there is suspicion of the intentional use of accelerants to initiate a fire^1^. Presence or absence of accelerants in fire site is a key factor in determining whether the fire is a case of arson. This usually have a positive significance for court trials and curbing crime^2–4^. In traditional approaches, detecting and identifying unexplained neat ignitable liquids in a fire scene, or recovering ignitable liquid residues from fire debris, and subsequent analysis of the sample by GC–MS (Gas Chromatography-Mass Spectrometry), IR (Infrared) spectroscopy, and UV–Vis (Ultraviolet–Visible) spectroscopy^5–9^. However, the success of this method is dependent on its ability to extract a pure sample for analysis. In fire sites, the destructive nature of a fire, together with the high temperatures, destroys and causes evaporation of the accelerant. Furthermore, fire debris samples are usually taken hours after the fire has been extinguished and during this delay they might be subjected to additional chemical modification processes. All of these phenomena can significantly modify the chemical composition and thus lead to erroneous conclusions^10^. The complex environments of the fire sites such as the oxidizing atmosphere produced by combustion of accelerants, high-temperature, carbon produced by incomplete combustion, plasma produced by flame, air convection caused by turbulence, and interaction of accelerants and metallic surfaces among other factors affect the formation of an oxide layer on metallic surfaces present in a fire scene^11,12^. Cognizant to this, the oxidation products on metallic surfaces in a fire site can give information of the fire site temperature, atmosphere composition and combustion time based on the high-temperature oxidation theory. Herein, the oxidation behavior of metallic materials present in the combustion atmosphere was used to judge the presence of the accelerants in a fire scene based on this theory. Previous studies demonstrated that the oxidation characteristics of carbon, steel, copper, aluminum alloys, and stainless steel materials may be closely related to the oxide properties alerted by the combustion byproducts in combustion environments^13–17^. Carbon produced during the combustion of accelerants can have a range of effects on formation of the oxide layer on metal surfaces and facilitates the inference of the type of accelerant present in the combustion environment. Cognizant to this, studying the carbon characteristics on metal surfaces can aid fire investigation. Herein, mild steel with a lower carbon content was used as the experimental material to exclude the interference of the carbon contained in the original metal material. The kerosene was used as the experimental accelerant because of its widespread use and flammability.

## Materials and methods

Table 1 shows the chemical composition of the mild steel materials employed in this study. The specimens were cut using a wire cutting machine into a sheet-like shape with gauge dimensions of 3 mm × 10 mm × 20 mm. Holes (2 mm in diameter) were then formed by punching on the top to facilitate hanging samples. In order to ensure that the initial state of the sample is consistent, the samples were polished with 600# ~ 2000# SiC (Silicon Carbide) sandpapers and then with diamond paste. They were then ultrasonically cleaning in acetone and then in absolute ethanol for 10 min in each. They were finally blow-dried using nitrogen and placed into the sample holder. The entire experiment was conducted to simulate a fire site environment accelerated by kerosene.

**Table 1:** Chemical composition of carbon steel (wt., %).

C	Si	Mn	P	S	Ni	Cr	Cu	Al	N	Fe
0.025	0.015	0.020	0.009	0.005	0.025	0.015	0.020	0.009	0.005	Bal

**Figure 1:**
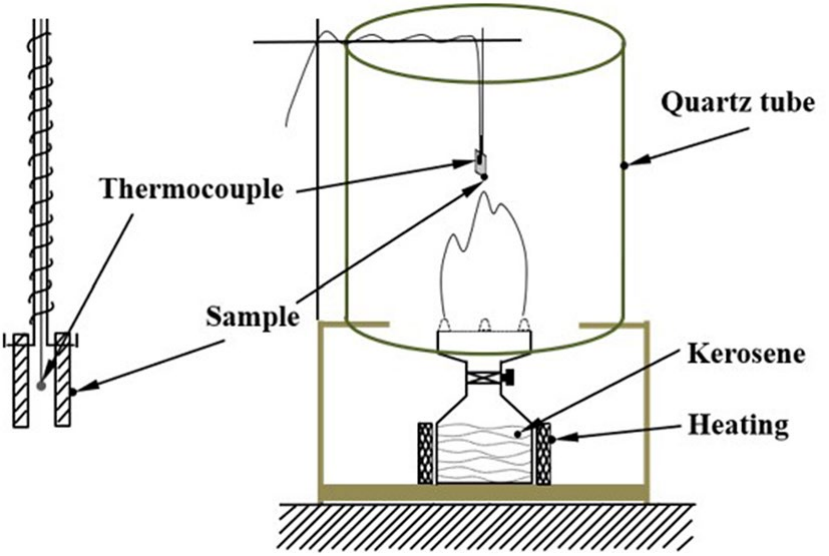
The device that simulated kerosene-combustion atmosphere.

**Figure 2:**
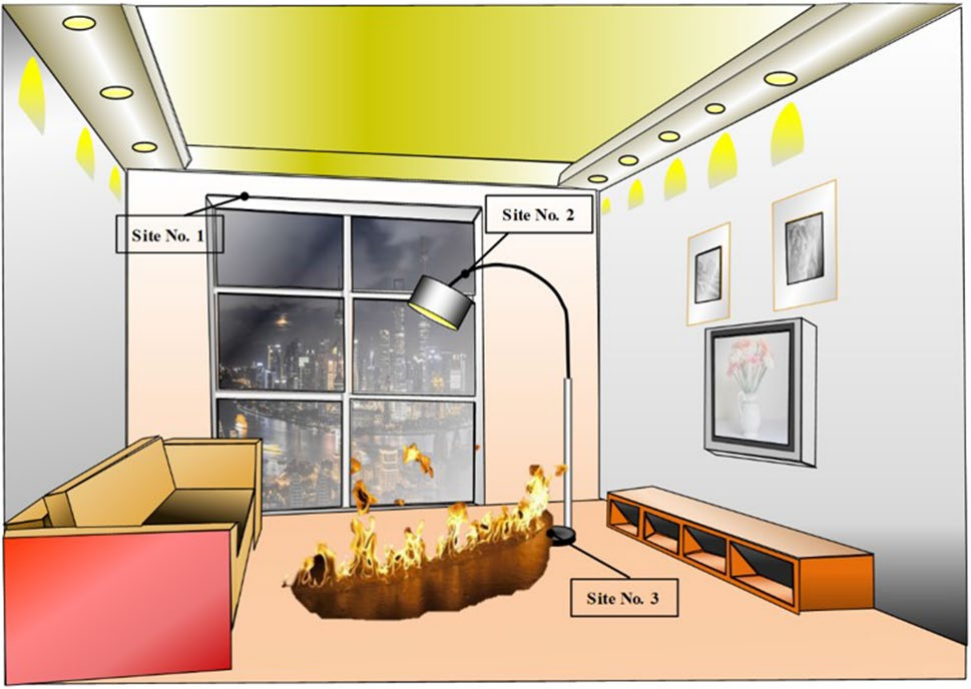
Schematic diagram of the classification of metal oxidation in the fire scene.

The experimental setup is sketched in Fig. 1. The oxidation behaviors of metallic materials in the fire scene were generally divided into three types based on different positions in the fire scene: (1) In the early stage of the fire, the oxidation caused by the kerosene spilled on the metal surface, (2) Near the flame, the metal material was oxidized by direct erosion of the flame, and (3) In areas not in direct contact with the flame, the metal material was oxidized by the combustion atmosphere in the fire scene; it was the main mode of metal oxidation in the fire (Fig. 2).

**Figure 3:**
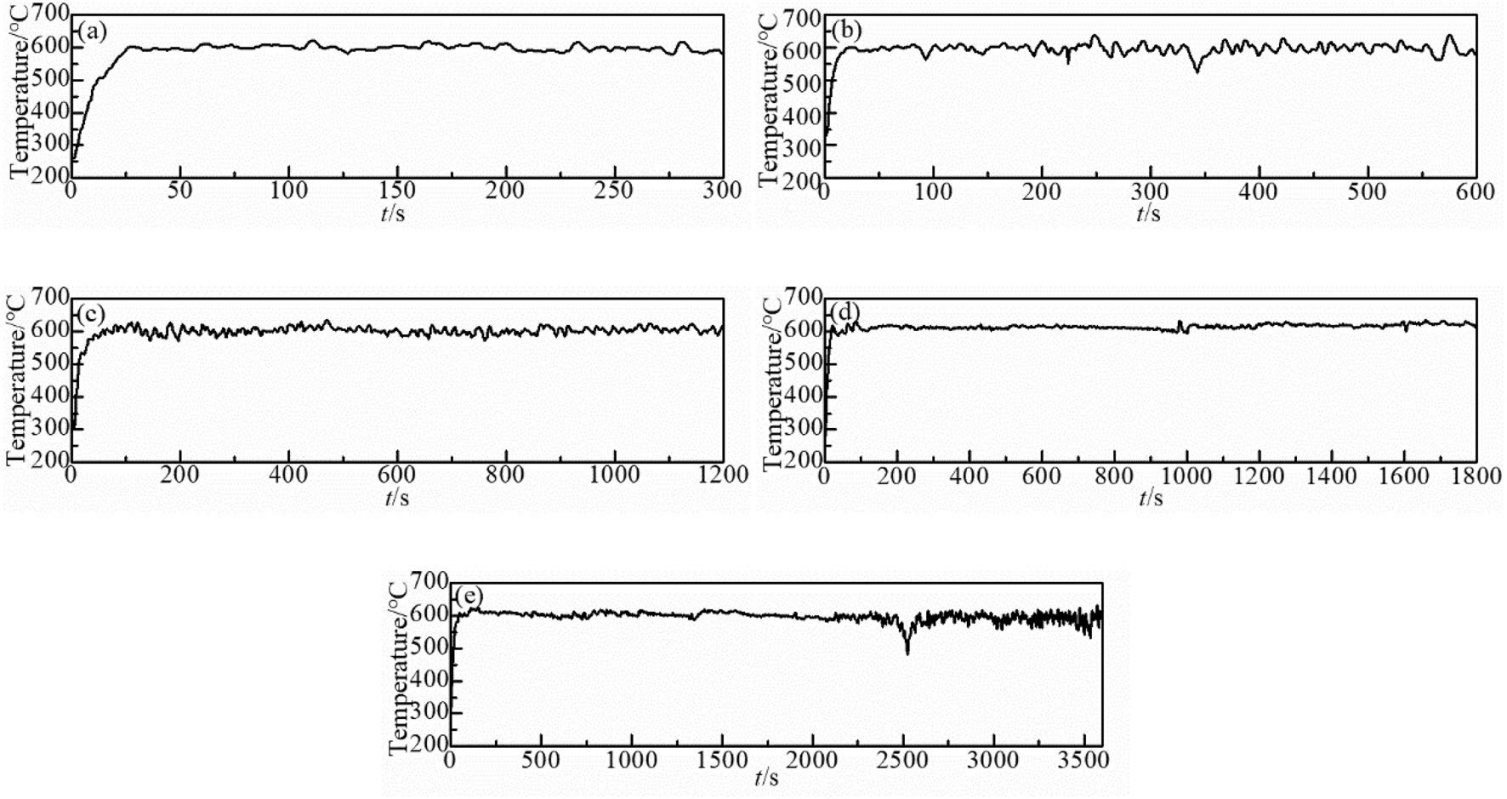
Temperature curve of oxidation time at 600 °C for (**a**) 5 min, (**b**) 10 min, (**c**) 20 min, (**d**) 30 min and (**e**) 60 min.

**Figure 4:**
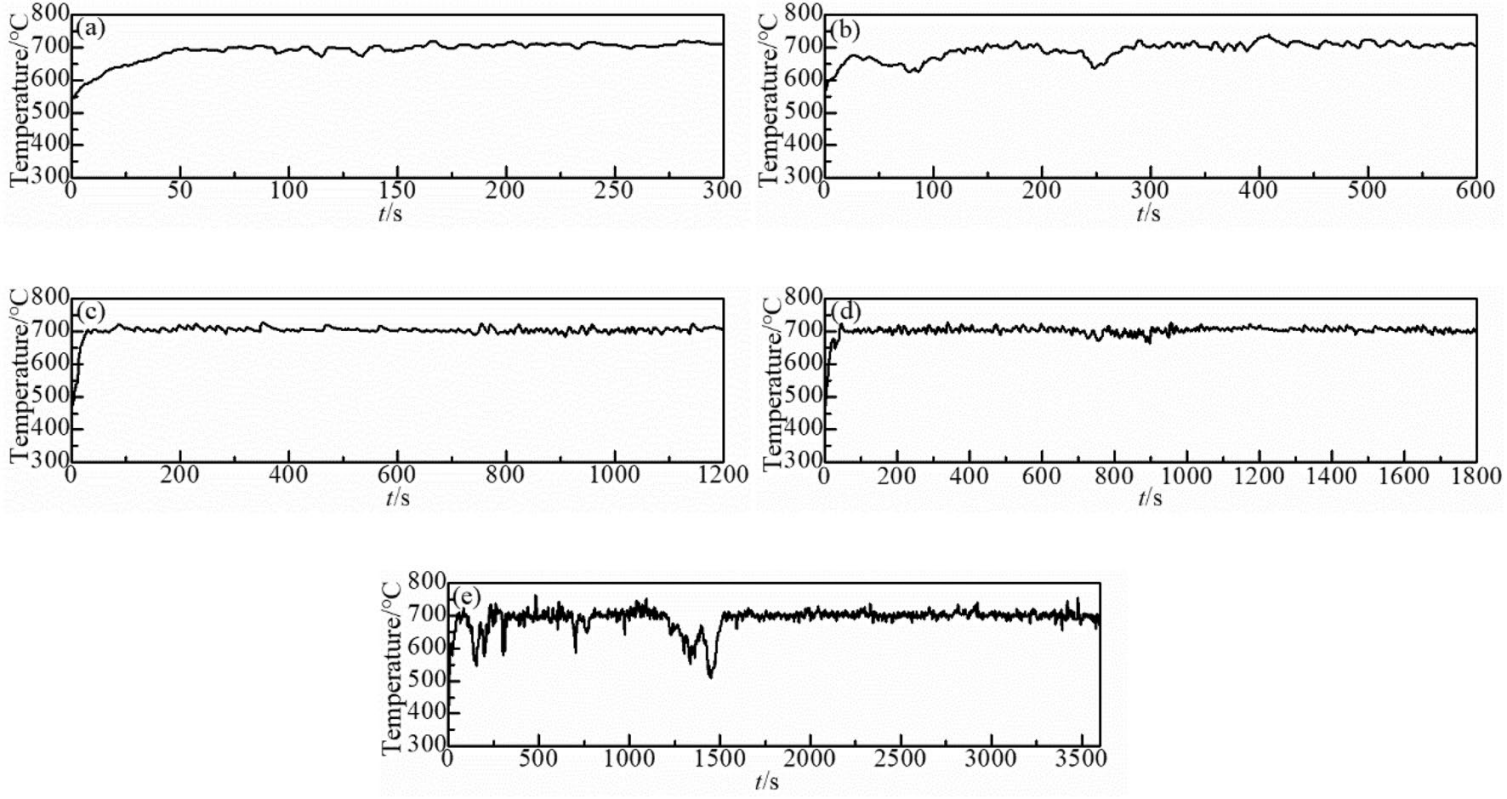
Temperature curve of oxidation time at 700 °C for (**a**) 5 min, (**b**) 10 min, (**c**) 20 min, (**d**) 30 min and (**e**) 60 min.

This study focused on the oxidation behavior of metals in the atmosphere of the fire site. The oxidation temperature was controlled by adjusting the flow rate of the kerosene. The temperature of the experimental trough was maintained between 600 °C and 700 °C while the oxidation times were performed for 5, 10, 20, 30, and 60 min. The oxidation temperature during burning was measured using a thermocouple (Figs. 3 and 4). At the end of the oxidation experiment, the samples were cleaned using ethanol to remove carbon particles attached to their surfaces. The samples were then weighed using a precision electronic balance (0.01 mg precision, BT255, Sartorius, Germany). Their lengths, widths, and height were measured using a vernier calipers (precision = 0.01 mm). The morphologies and cross-sectional morphology of the samples were then characterized using a field emission scanning electron microscope (SEM, Leo 1530 FEG SEM, Carl Zeiss SMT Ltd) at an accelerating voltage of 20 kV. The phase composition of the oxide layer was analyzed using energy dispersive X-ray spectrometer (INCA EDS, Oxford Instruments) and X-ray diffractometer (XRD-6100, Shimadzu). Elemental analysis and identification of the oxidation states were studied by X-ray photoelectron spectroscopy analysis (XPS, ThermoFische, ESCALAB Xi + , America).

## Results

### Surface morphology analysis

**Figure 5:**
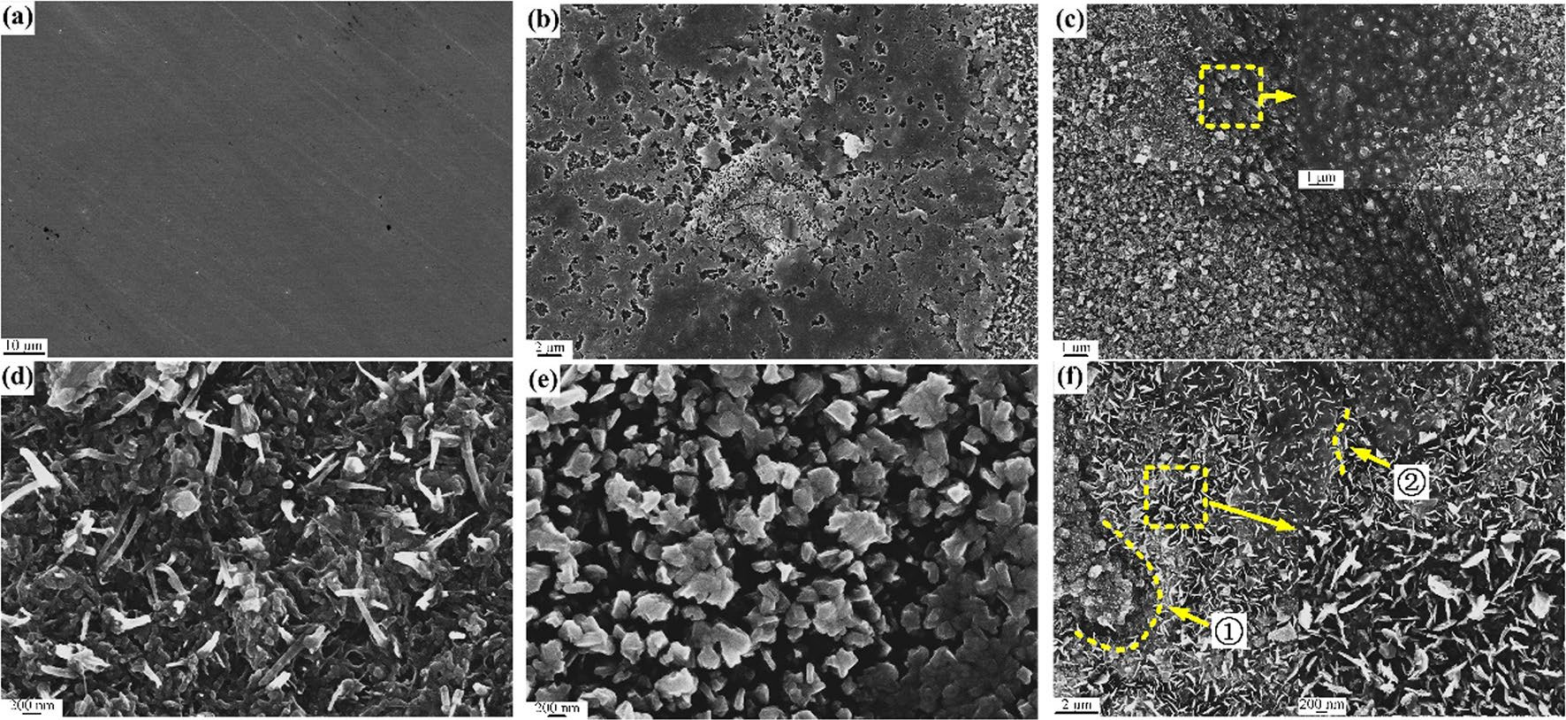
SEM surface morphology of the samples after oxidation at 600 °C for (**a**) 0 min, (**b**) 5 min, (**c**) 10 min, (**d**) 20 min, (**e**) 30 min, and (**f**) 60 min.

The surface morphology of the metal was observed using SEM when the oxidation temperature was about 600 °C (Fig. 5). Oxidation for 5 min led to the formation of holes on the surface and uneven growth of oxide particles (Fig. 5b) compared with the unoxidized surface (Fig. 5a). This revealed that presence of multiple oxidizing atmospheres in the kerosene combustion environment greatly accelerated the oxidation of the metal. The unevenness oxidation of the metal maybe caused by the temperature instability in a fire scene. The oxides started to grow and break through the epidermis when oxidized for 10 min with the extension of time (Fig. 5c). A cracked surface oxide layer formed a needle-shaped oxide that got attached to the metal surface (Fig. 5d). This revealed that both Fe^n+^ outward and inward transfer oxidation mechanisms occurred during oxidation. When the oxidation time was extended to 30 min, the needle-shaped oxide on surface was replaced by a granular oxide (Fig. 5e). This was an indication that the oxide had grown and consistent with the XRD results (Fig. 8). In addition, there were numerous holes on the metal surface which were attributed to the inward transport of large amounts of oxidizing components in the combustion atmosphere. Catastrophic oxidation occurred on the surface of the sample when the oxidation time was increased to 60 min, which may be due to the synergistic oxidation system composed of strong oxidizing components in the combustion atmosphere greatly accelerate the oxidation speed, oxide grew on the No. 1 areas, and the No. 2 areas indicated that the oxidation led to formation of a granular oxide (Fig. 5f). This further illustrated that the metal oxidation was non-uniform because of instability of the temperature in the fire scene. The EDS (Energy Dispersive Spectroscopy) analysis results of samples further revealed that about 10% of carbon was deposited on the surfaces. The carbon content (12% -13%) of the surface oxide in the un-burnt surface was generally higher than that of other locations. This strongly suggested that at the initial stage of oxidation, the carbon generated by the accelerants was incompletely burnt and deposited on the surfaces. As the oxidation proceeded, the outermost layer was gradually torn by oxide and shapes like needle formed.

**Figure 6:**
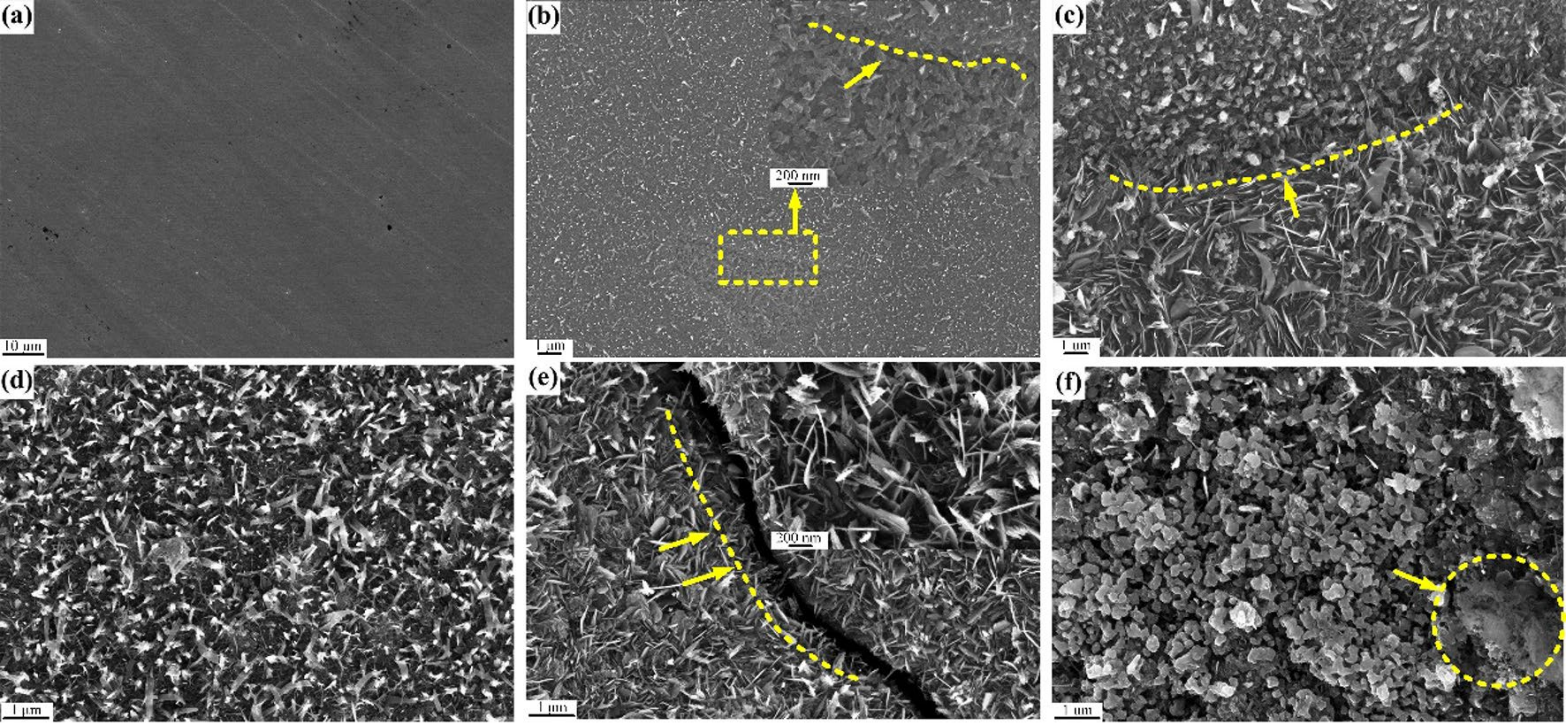
SEM surface morphology of the samples after oxidation at 700 °C for (**a**) 0 min, (**b**) 5 min, (**c**) 10 min, (**d**) 20 min, (**e**) 30 min, and (**f**) 60 min.

Figure 6 shows the sample surface morphology when the temperature rose to 700 °C. After 5 min, an oxide layer of a certain thickness appeared on the sample surface (shown by the arrow in Fig. 6b), compared with the unoxidized surface (Fig. 6a), the oxidation was more serious compared to 600 °C, which was associated with the higher oxidation temperature. In addition, both needle shaped and granular oxides on the surface showed there was uneven oxidation on the surface of the sample (Fig. 6c). This was attributed to the instability of the oxidation in the fire, which was different from the usual constant temperature oxidation. The needle-shaped oxides also appeared on the surface of the sample because of tearing of the inner oxide by the outer oxide layer (Fig. 6d,e). These oxidation was more severe than at 600 °C and caused the oxide layer to crack (Fig. 6e). It was related to the higher oxidation temperature as well as exposed to oxidizing atmospheres. When the oxidation time was extended to 60 min, most of the oxides gradually changed from needle-like growths to particles. Nonetheless, the oxides continued to grow (Fig. 6f), which agrees with the 600 °C surface oxidation, both of them suffered from catastrophic oxidation.

**Figure 7:**
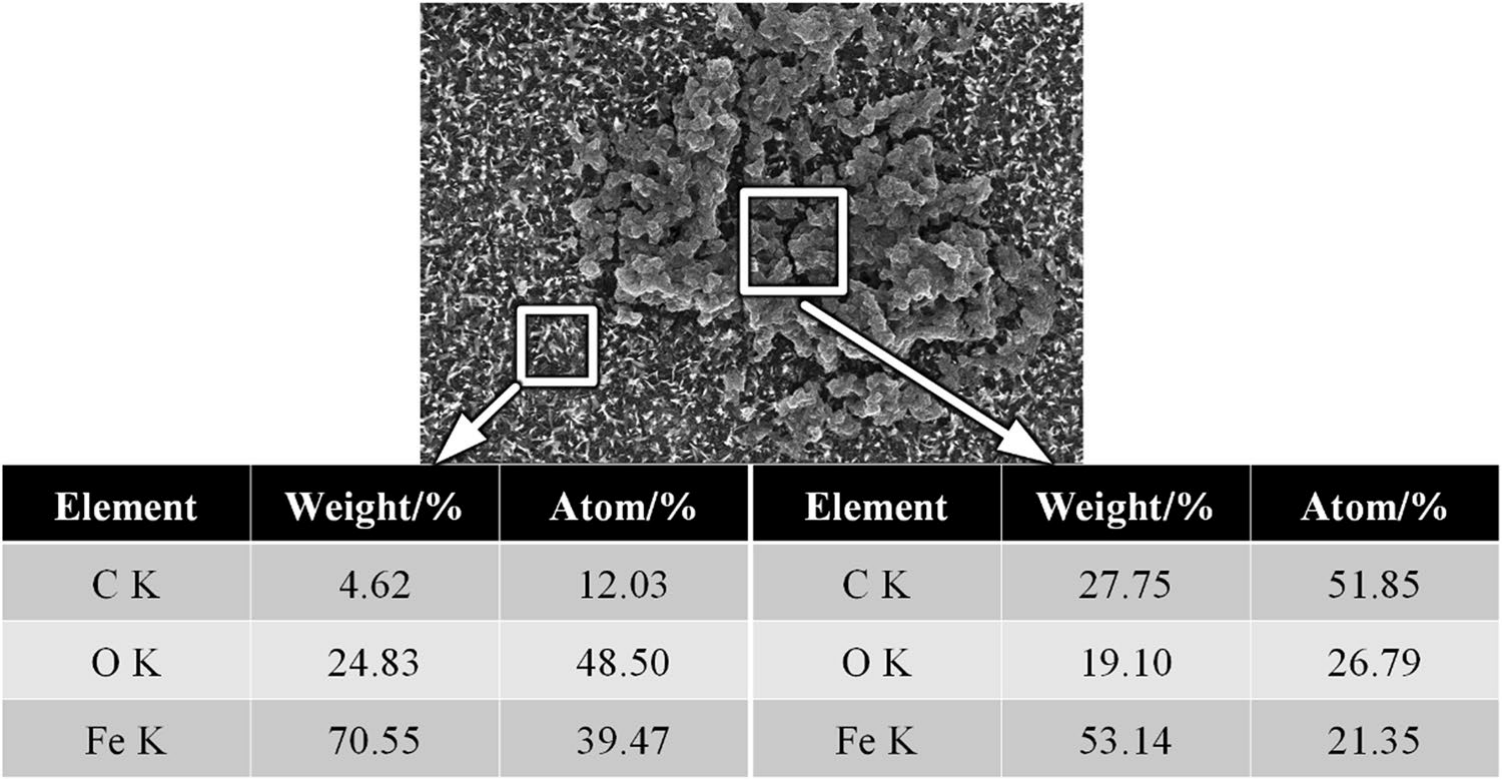
Schematic diagram of carbide element content distribution on the sample surface oxidized at 700 °C for 20 min.

Similarly, EDS results showed that about 10% carbon was deposited on the sample surface. This was attributed to incomplete combustion of the accelerants. The carbon content deposited by kerosene combustion was higher than the carbon content deposited on the metal surface by ethanol combustion. This was attributed to the different lengths of the carbon chain of the accelerants^13^. Additionally, carbides with high carbon content were found on the surface of the sample after an oxidation time of 20 min (Fig. 7), which was formed after a mixture of the carbon and oxides deposited on surface. This was assumed to be an "oxide–carbon" symbiosis model which needed further studies to decipher its mechanism.

### Oxidation layer phase analysis

**Figure 8:**
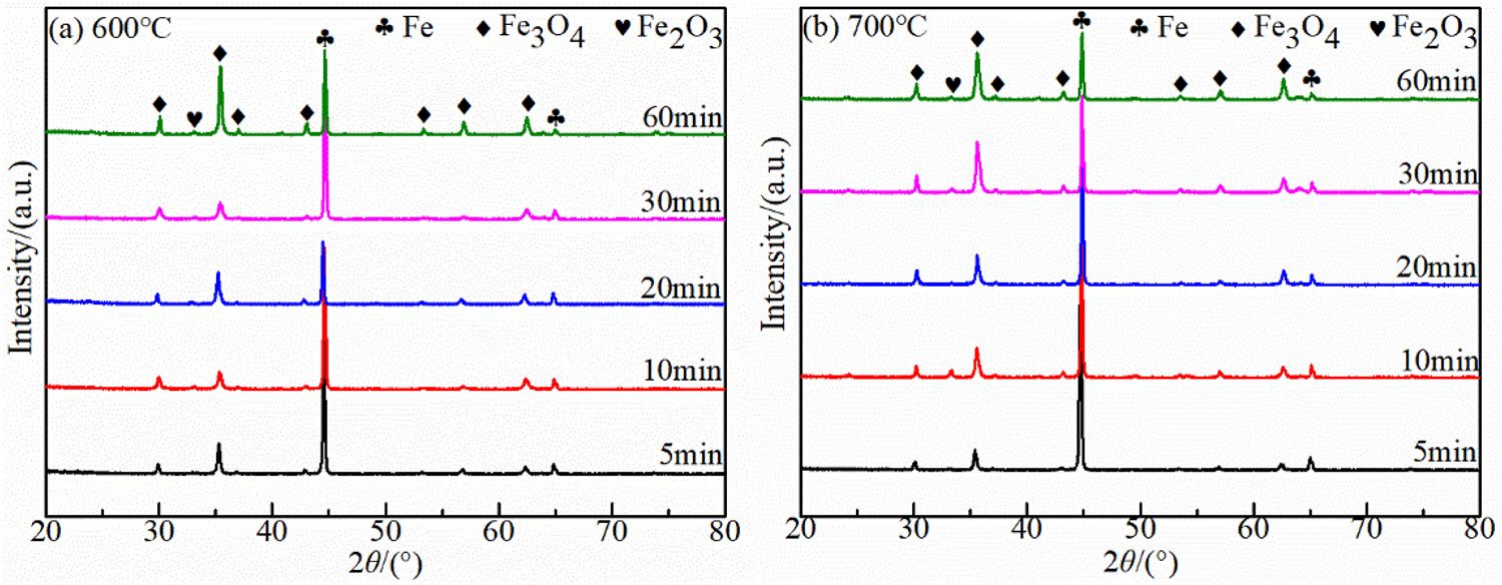
X-ray diffraction results of mild steel under kerosene oxidation atmosphere at (**a**) 600 °C and (**b**) 700 °C.

**Figure 9:**
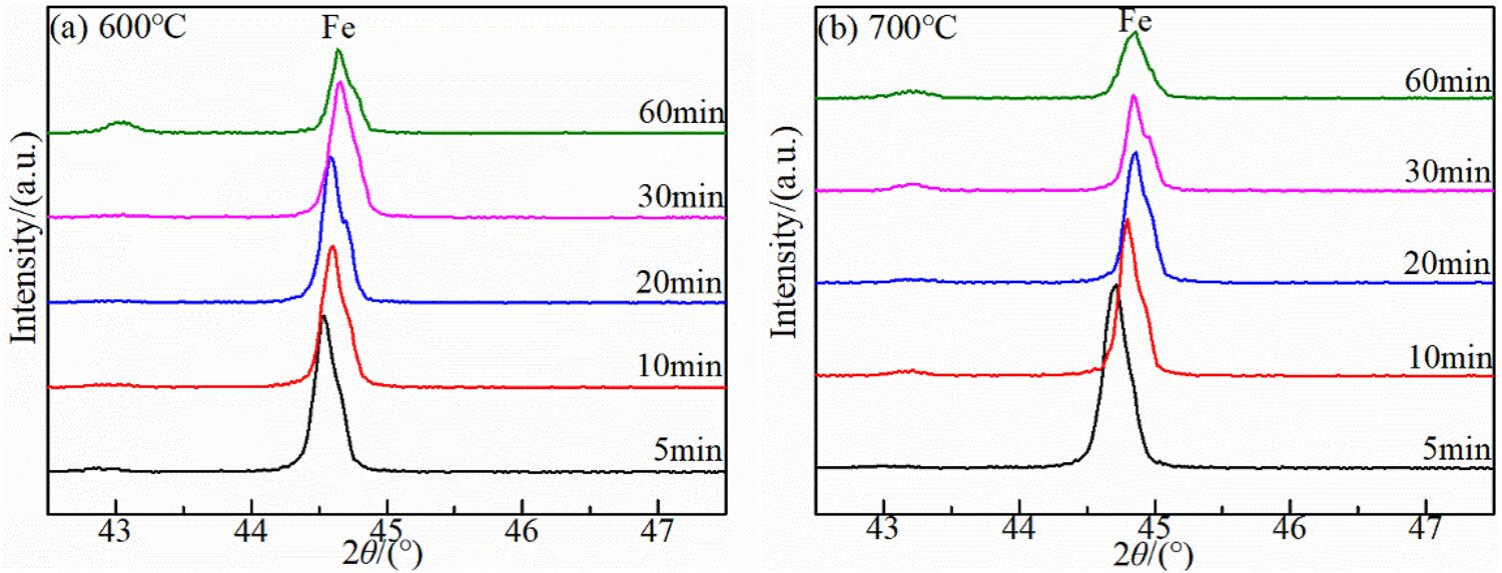
The 43°–47° X-ray diffraction results of mild steel under kerosene oxidation atmosphere at (**a**) 600 °C and (**b**) 700 °C

The XRD patterns at 600 °C and 700 °C after different oxidation times revealed that the Fe_3_O_4_ phase increased with increasing oxidation time (Fig. 8) and the hematite (Fe_2_O_3_) phases were detected at the late stage of oxidation in both temperature. However, the wustite (FeO) phase was not detected. The temperature was relatively stable in this experiment, which can be ruled out unstable oxidation temperature prevents wustite (FeO) from nucleating and growing. This could be related to strong oxidation of the combustion atmosphere, which promotes further oxidation of divalent iron. It was different from oxidation of carbon steel in hot air. Figure 9 showed the Fe peak of the matrix around 2θ = 44.5° was right deviation. The results further revealed all the iron-based peaks around 2θ = 44.5° had a right-biased trend when the oxidation temperature were 600 °C and 700 °C. It was assumed that the carbon deposited during the oxidation process penetrated into oxide layer thereby causing distortion of the Fe lattice, which caused the Fe peak shifting to right.

### The effect of carbon in the combustion atmosphere on the oxide layer

**Figure 10:**
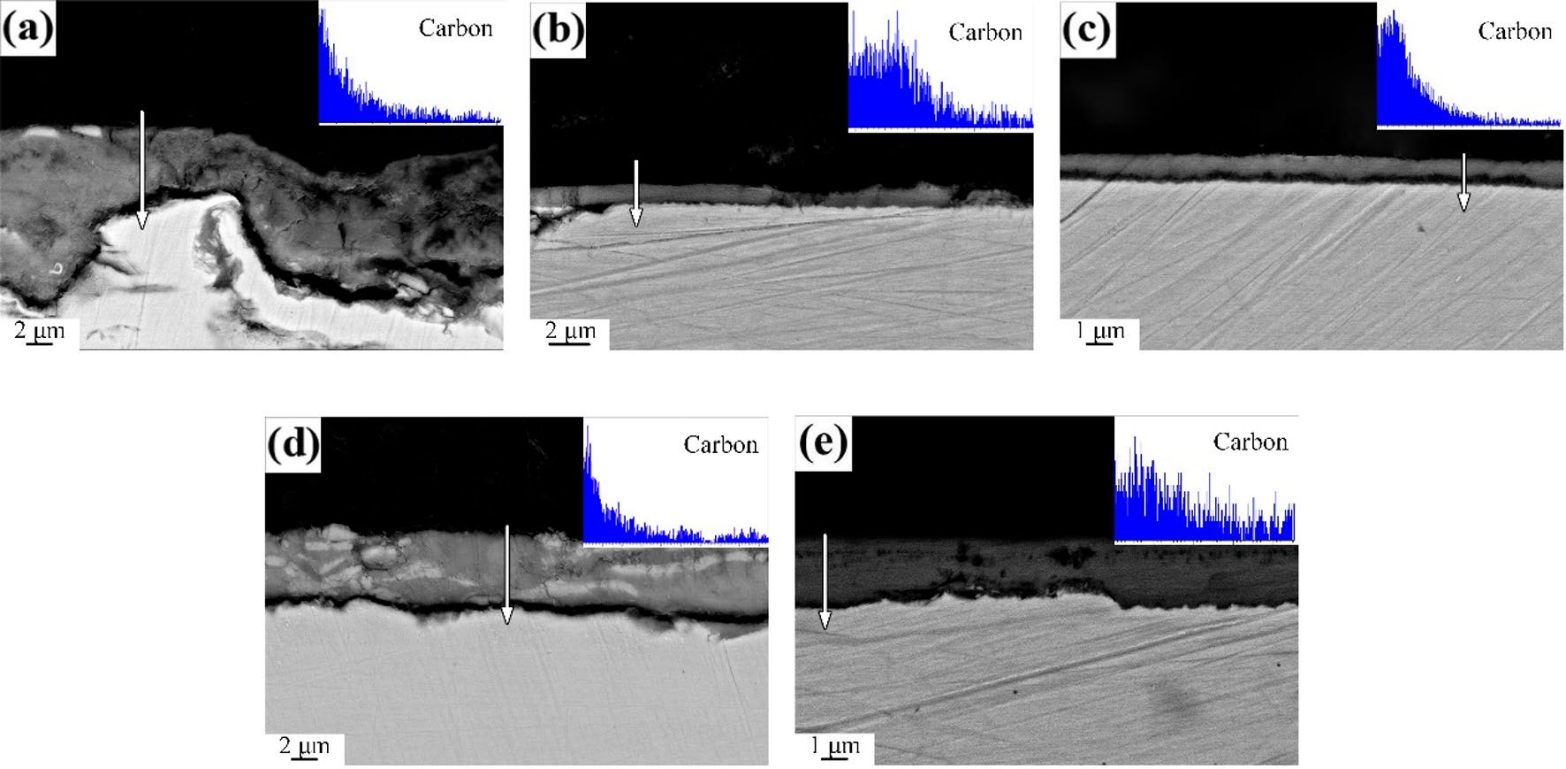
Sectional morphologies of mild steel specimens after oxidation at 600 °C for (**a**) 5 min, (**b**) 10 min, (**c**) 20 min, (**d**) 30 min and (**e**) 60 min.

**Figure 11:**
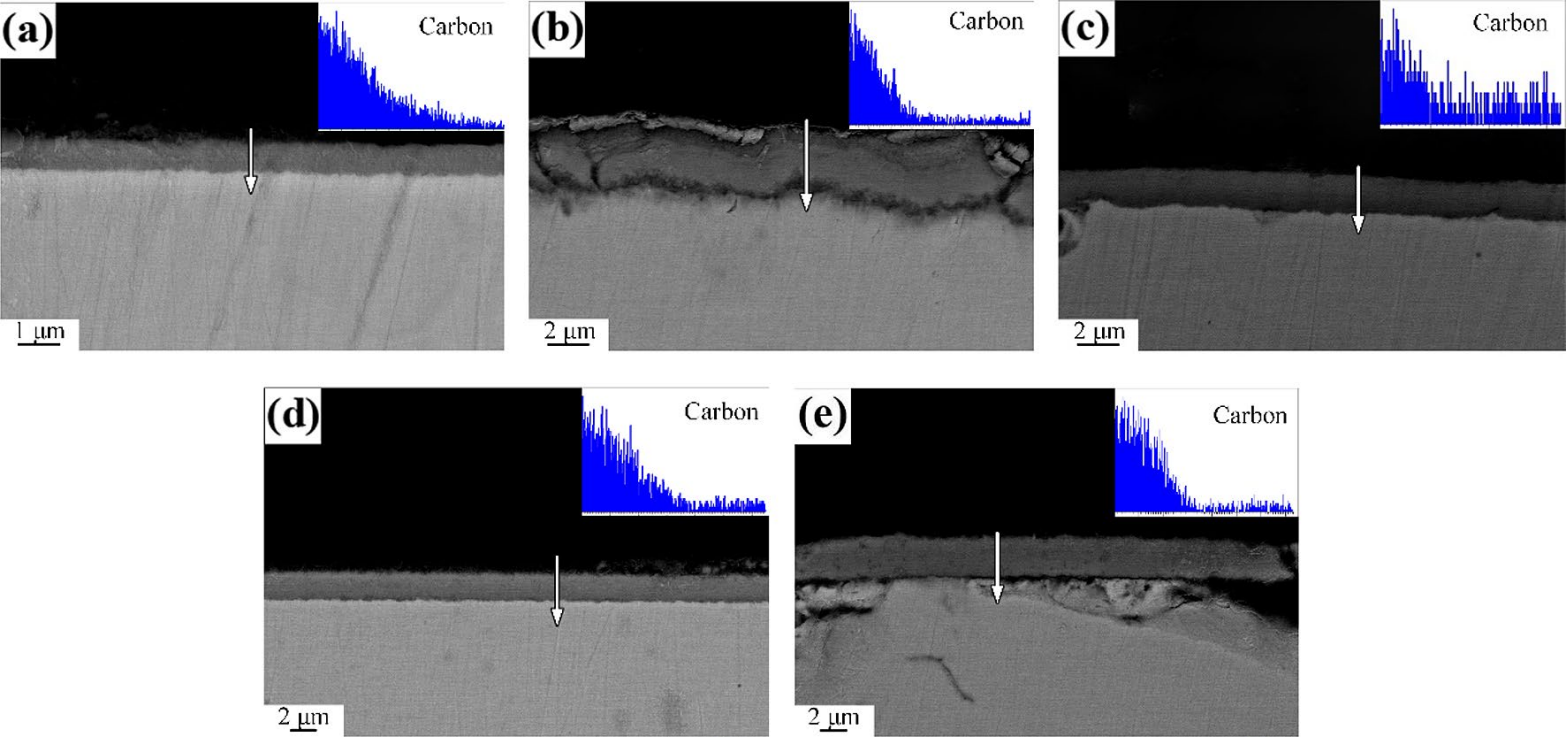
Sectional morphologies of mild steel specimens after oxidation at 700 °C for (**a**) 5 min, (**b**) 10 min, (**c**) 20 min, (**d**) 30 min and (**e**) 60 min.

Figures 10 and 11 were the cross-sectional SEM and EDS line scan results of mild steel oxidized under kerosene combustion atmosphere at 600 °C and 700 °C, respectively. carbon dioxide (CO_2_), carbon monoxide (CO) and other carbon-containing components under the kerosene combustion atmosphere had a carburizing effect on the oxide layer. The element line scan results showed that the carbon content of the oxide layer decreased from outside to inside. The results indicated that carbon in combustion atmosphere had entered the oxide layer. It was consistent with the overall shift right of Fe peak in the XRD. As such, they strongly suggested that carburization caused lattice distortion thereby resulting in a right-biased Fe peak in XRD. However, there was no defined trend for the thickness of the oxide layer at various temperatures and oxidation times. This was attributed to the uneven oxidation caused by the instability of the oxidation temperature.

**Figure 12:**
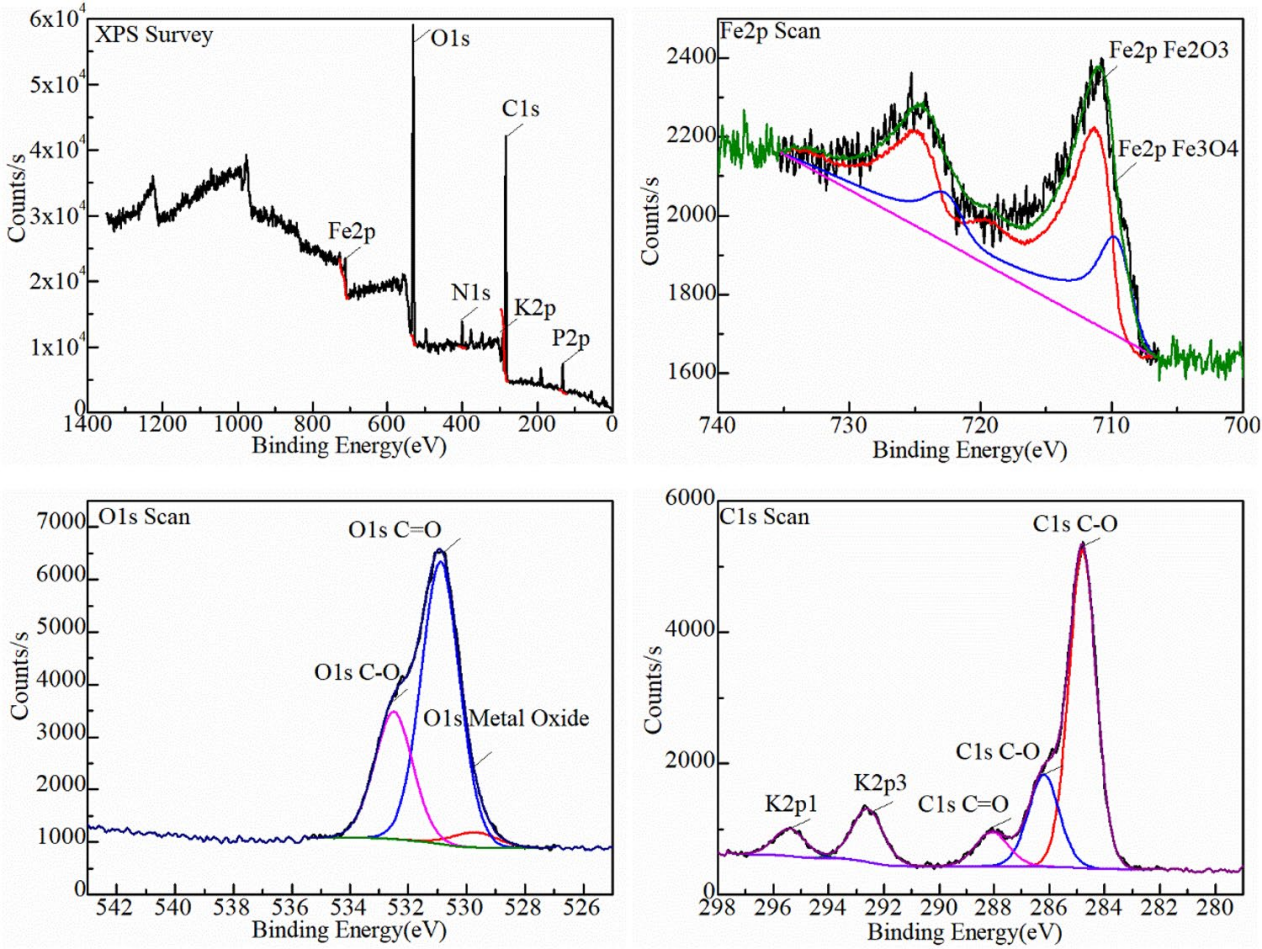
XPS analysis results of the surface of mild steel oxidized under kerosene combustion atmosphere at 600 °C for 60 min.

**Figure 13:**
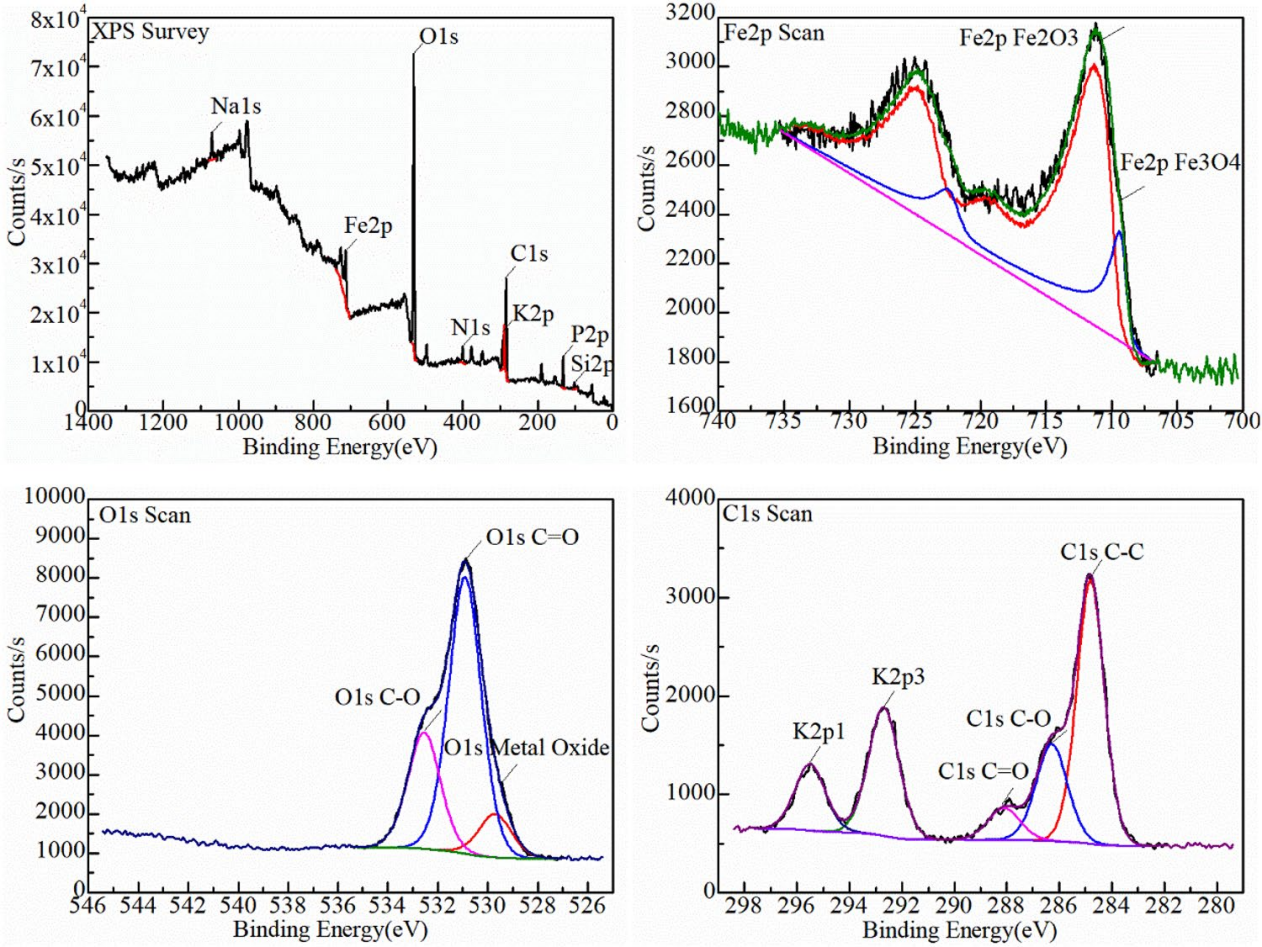
XPS analysis results of the surface of mild steel oxidized under kerosene combustion atmosphere at 700 °C for 60 min.

XPS was used to analyze the oxidized surface to further determine how the carbon in the combustion atmosphere penetrated the oxide layer and what form it existed in the oxide layer (Figs. 12, 13). The full XPS spectrum revealed the oxidation scale mainly contained Fe, O, C, K, N, P elements, and Fe, O, C were the most abundant. These elements were attributed to the kerosene composition. C mainly existed in the chemical states of organic carbon C–C, C–O, and C=O. In the same line, no metal–carbon peak was found near the electron binding energy of 282 eV.This indicated that carbon did not enter the Fe lattice and further explained the growth mechanism of carbon and oxide symbiosis. The main forms of oxygen element were C–O and C=O in organic matter and metal oxide. The ratio of hematite (Fe_2_O_3_): magnetite (Fe_3_O_4_) in the two sets of temperatures increased from 2.17:1 at 600 °C to 4.04:1 at 700 °C. This strongly indicated that higher temperature directly accelerated the oxidation of the sample. Moreover, no satellite peak of FeO appeared, it corresponded to the XRD results.

The relative content of chemical-bonding state of the elements was shown in Table 2. An increase in the oxidation temperature led to a decrease in the C–C content. However, the contents of the three forms of oxygen increased (Table 2). It was attributed to part of the carbon forming CO_2_ or CO detaching from the surface or transforming into an organic carbon. Nevertheless, the Fe^3+^ content increased because of higher oxidation temperature.

**Table 2:** The comparison table of relative contents of chemical-bonding state of elements.

Name	600℃		700℃	
	Peak BE	Atomic/%	Peak BE	Atomic/%
C1s C–C	284.8	40.86	284.8	25.33
C1s C–O	286.2	13.4	286.28	10.62
C1s C=O	288.06	5.38	288.08	3.62
Fe2p Fe_2_O_3_	711.3	2.24	711.35	5.02
Fe2p Fe_3_O_4_	709.75	1.03	709.4	1.24
O1s C–O	532.49	10.3	532.55	13.27
O1s C=O	530.89	22.8	530.91	31.49
O1s metal oxide	529.7	1.45	529.7	4.95

## Discussions

### The mechanism of carbon on the oxide layer

Kerosene is a mixture of high-boiling hydrocarbons with C11–C17 carbon atoms, and small amounts of impurities such as sulfides and colloids. During kerosene combustion, the hydrocarbons are first cracked to form carbon and hydrogen, and then interact with free oxygen atoms to form H_2_O and CO^18,19^. Under the condition of sufficient oxygen, the CO will continues to react with oxygen molecules to produce CO_2_, however, the indoor environment increase the probability of incomplete combustion. These can be thermodynamically determined that there is carbon deposition in kerosene combustion. During the deposition process, elemental carbon tends to deposit at high surface energy or to agglomerate as observed in Fig. 7^20^. The amount of the deposited carbon may be related to the length of the carbon chain of accelerants. The longer the carbon chain, the higher the carbon content deposited on the sample surface^13^. Deposition of carbon occurred inside the oxide layer through an "oxide–carbon" symbiosis model and does not enter the crystal lattice of substrate metals.

The form of carbon in the oxide layer is closely related to the environment. In general, the carbon deposited during combustion have three forms: (1) enter the crystal lattice of the metal to form a solid solution, (2) forming amorphous carbon on surface such as carbon black, and (3) forming elemental carbon in the oxide layer. The form of carbon deposited on metal surface is determined by the accelerants combustion atmosphere, types of metal and the temperature in the fire scene^21–24^. The carbon in the accelerants have different effects on the formation of metal oxides. Accelerants can be derived by analyzing the form of carbon deposited on the metal surface combined with the temperature and metal in fire scene. The CO_x_-containing atmosphere formed because of combustion increases the carbon potential in the atmosphere thereby forming a semi-infinite diffusion couple with the metal. The couple is conducive to gas carburization as illustrated in Figs. 10, 11^25^. The functional group elements (such as S, N, Cl, etc.) in the accelerants also penetrates the oxide layer during the carburization process to form characteristic components such as metal nitrides, metal chlorides, and metal sulfides as illustrated in Figs. 12, 13^26^. Evidently, analyzing the form of carbon in the oxide layer in fire scene is valuable in fire investigations. Nevertheless, this should be explored further to identify the specific mechanisms involved.

### The mechanism of combustion atmosphere on the oxide layer

Oxidizing components such as CO_2_, CO, H_2_O, and SO_2_ produced by kerosene combustion also form a synergistic oxidation system independently or with air to accelerate the oxidation and flaking of metal materials.

The metal reacts with H_2_O to generate hydrogen which is subsequently absorbed by the metal at high temperature to accelerate oxidation (Reaction 1)^26^.

1 $${\text{M}} + {\text{H}}_{{2}} {\text{O}}\left( {\text{g}} \right) = {\text{MO}} + {\text{2H}}\left( {{\text{soln}}} \right)$$

**Figure 14:**
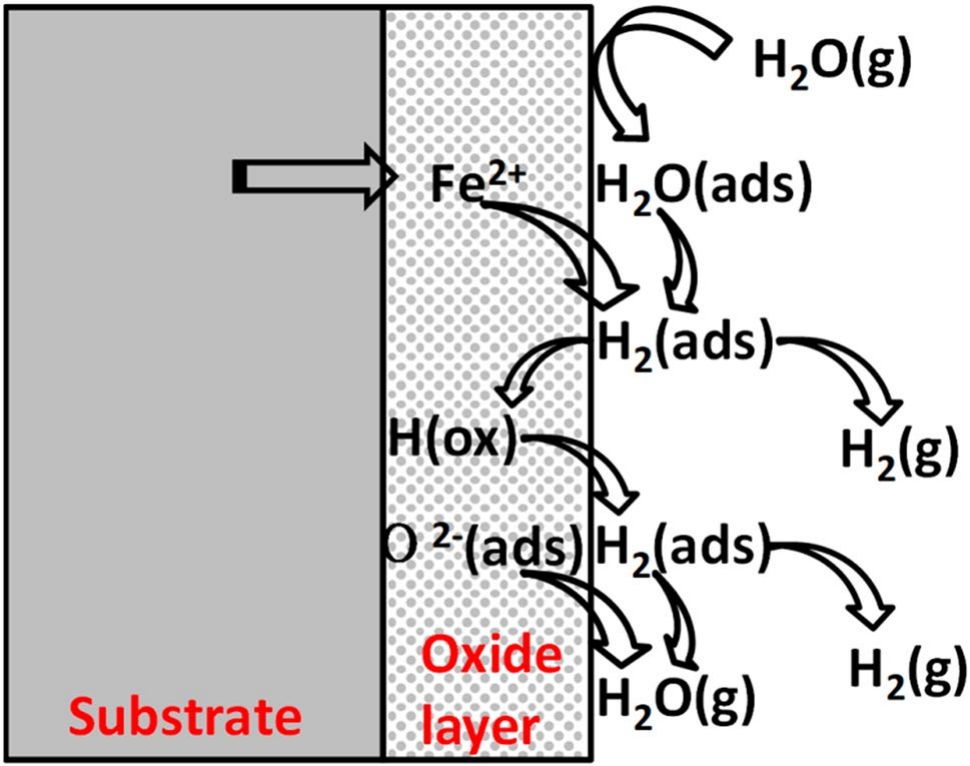
Water vapor dissociation-adsorption process on metal surface.

And there is a dissociation-adsorption process of water vapor in the air-H_2_O oxidation atmosphere (Fig. 14)^27,28^. H_2_O_(g)_ forms H_2_O_(ads)_, and then H_2_O_(ads)_ reacts with metal ions to generate H_2(ads)_. There are two reaction pathways for H_2(ads)_: desorption into the air and dissolution into oxide to form H_(ox)_. Desorption into the air causes the oxide layer to crack and fall off (is consistent with the SEM results) while dissolution into oxide to form H_(ox)_ promotes diffusion of ions in the oxide layer thereby accelerating oxidation of metals^29^.

The "air-CO_2_-CO" redox atmosphere promotes oxygen transport through pores in the oxide layer structure. There is an equilibrium reaction (Reaction 2) in the holes because the oxygen’s partial pressure on the holes in the oxide layer is lower than that in the metal surface^30^.

2 $$\mathrm{CO}2\left(\mathrm{g}\right)=\mathrm{CO}\left(\mathrm{g}\right)+\frac{1}{2}\mathrm{O}2(\mathrm{g})$$

The generated oxygen reacts with the metal matrix through the oxide layer while the CO reduces the oxide generated on the metal surface thereby leading to generation of internal stress which causes the oxide layer to peel off. This promotes Reaction 2 to the right, CO_2_ as an oxidizing atmosphere can also directly react with metals (Reaction 3)^26^.

3 $${\text{Fe}}\left( {\text{s}} \right) + {\text{CO}}_{{2}} \left( {\text{g}} \right) = {\text{FeO}}\left( {\text{s}} \right) + {\text{CO}}\left( {\text{g}} \right)$$

However, the oxidizing components in the combustion atmosphere promoted further oxidation of Fe^2+^ to form a Fe_3_O_4_ phase instead of a FeO phase. This result was contrary to the composition ratio of the oxide layer in which the FeO component occupies most of the oxidation layer in the conventional hot air, which also contributes to fire investigation. Herein, the oxide layer occupied most of the Fe_3_O_4_ phase which had defects in the octahedral and tetrahedral positions because of its reverse spinel structure (Fe^2+^ occupying octahedral positions, half of Fe^3+^ occupying tetrahedral positions). And the iron ions were controlled to diffuse out of the Fe_3_O_4_ layer through these two positions for further oxidation. Although the growth of oxide layer mainly depends on the outward diffusion of iron ions, SO_x_ in the combustion atmosphere can be transported inwards along physical defects in the oxide layer^14,26,31^. It is the combination of multiple oxidizing atmospheres that leads to the bidirectional oxidation of the sample: iron ions are transported outwards while the oxidative atmosphere is transported inwards, which significantly accelerates the oxidation attack on the metal compared with traditional oxidation in hot air.

## Conclusion

This paper proposes a new method which uses the oxidation behavior of metal materials in the fire atmosphere to judge the existence of accelerants. The oxidation behavior of mild steel in the combustion environment of kerosene accelerants is studied. From this study some useful conclusions are summarized as follows.

1. The mild steel is oxidized under the kerosene combustion atmosphere thereby causing carbon deposits to appear on the surface, and the content may increase with the increase of the carbon chain of the accelerants, which is attributed to the incomplete combustion of kerosene. meanwhile, the carbon in the oxide layer is mostly organic carbon which are the main components of accelerants.
2. The carbonaceous atmosphere generated by the combustion of the hydrocarbon accelerator has a carburizing effect on the metal oxide layer. The results show that the carbon did not enter the Fe lattice, and it is speculated that carbon and oxides exist in the form of "oxide–carbon" symbiosis.
3. Various oxidizing atmosphere composite systems by the accelerator promote the formation of metal oxide layers. The bidirectional oxidation mode in the oxide layer further accelerates the oxidation rate. At the same time, the FeO phase is not found in the oxide layer because of the strong oxidation of the combustion atmosphere.

A single method is not enough to make scientific conclusion in analysis of a complex system like fire. Combining characterization of surface scale with traditional chemical analysis of recovering ignitable liquid residues from fire debris are expected to offer complementary insight into fire characteristics, such as whether liquid accelerant is involved.
